# Effect of Different Exercise Modalities on Oxidative Stress: A Systematic Review

**DOI:** 10.1155/2021/1947928

**Published:** 2021-02-11

**Authors:** Anand Thirupathi, Meizi Wang, Ji Kai Lin, Gusztáv Fekete, Bíró István, Julien S. Baker, Yaodong Gu

**Affiliations:** ^1^Faculty of Sports Science, Ningbo University, Ningbo 315211, China; ^2^Savaria Institute of Technology, Eötvös Loránd University, Szombathely 9700, Hungary; ^3^Faculty of Engineering, University of Szeged, Szeged, Hungary; ^4^Centre for Health and Exercise Science Research, Department of Sport, Physical Education and Health, Hong Kong Baptist University, Hong Kong 999077, China

## Abstract

Exercise-induced benefits are being increasingly recognized in promoting health and preventing diseases. However, initial adaption to exercise response can have different effects on cells, including an increase in the formation of oxidants and inflammatory mediators that ultimately leads to oxidative stress, but this scenario depends on the exercise type and intensity and training status of the individual. Therefore, we aimed to understand the effect of different types of exercise on oxidative stress. Indeed, exercise-induced minimum oxidative stress is required for regulating signaling pathways. According to the Preferred Reporting Items for Systematic Reviews and Meta-Analyses (PRISMA) statement, a search for relevant articles was carried out on PubMed/Medline, ISI Web of Science, and Google Scholar using a broad range of synonyms such as oxidants, reactive oxygen species (ROS), oxidative stress, exercise, physical training, aerobic exercise, and strength exercise until 2019. This study selected a total of 18 articles for assessing the oxidative damage using various parameters such as malondialdehyde (MDA), protein carbonyl (PCO), and F1-isoprostanes and enzymatic antioxidants. We observed that any type of exercise can increase the oxidative damage in an exercise type and intensity manner. Further, the training status of the individual and specific oxidative damage marker plays a crucial role in predicting earlier oxidative damage in the exercise condition. However, some of the studies that we included for review did not perform follow-up evaluations. Therefore, follow-up programs using larger numbers need to be performed to confirm our findings.

## 1. Introduction

Life expectancy is associated with several factors, and physical exercise is one of the factors that help to promote human life expectancy. Studies have shown that exercise can prevent several life-threatening diseases such as cardiovascular diseases, obesity-related diseases, and some types of cancers [1–3]. Reactive oxygen species (ROS) can be the direct factor or associated factor to cause or prevent these diseases [4]. Research advancements in both exercise and free radical biology have been providing substantial developments in the knowledge related to the mechanisms of exercise and oxidative stress [5, 6]. Although initial investigations reported the negative effect of ROS, recent studies have shown that exercise-induced ROS can upregulate several enzymatic and nonenzymatic antioxidants in the biological system [7, 8], and exercise could be an optimizer of ROS in negating oxidative damage in the cells, while ROS can regulate signaling or act as a signaling molecule to muscular adaption. In this regard, several studies have concluded that regular physical exercise does not culminate in chronic oxidative stress in the active muscles [9, 10]. This supports the concept of exercise-induced hormesis. The term hormesis is used to define the biphasic dose-response curve in the biological system where a smaller amount of stress can facilitate adaptation whereas chronic and/or high dose of stressor can increase the damage to the cells [11, 12]. This scenario can play several regulatory roles from genotypic to phenotypic levels. However, this hormesis effect depends on the exercise type and intensity which could be the preserver or spoiler of this condition, and this can demand additional research.

The first study suggests that aerobic exercise can increase oxidative stress [13]. After this, several studies have reported that acute aerobic exercise increases oxidative stress and decreases antioxidant levels [5, 14, 15]. However, certain studies have shown that exercise was observed to influence only some oxidative stress markers or none at all [16, 17]. Also, studies have shown that anaerobic exercise can increase oxidative stress and decrease or increase antioxidants [18]. Further, whole-body resistance exercise increased oxidative damage. For example, resistance exercise at a 10-repetition maximum load increases the MDA level in the blood [19]. Furthermore, local resistance exercise, which is a single type of resistance training in a specific muscle group, can increase oxidative damage. In contrast, other studies have shown that resistance exercises did not cause oxidative stress, and this may be due to the training status of the individual [20, 21]. However, changes in the individual variability such as exercise-induced reductive stress or negligible stress have a considerable effect on some people. For example, oxidative stress response is changed in individuals who performed eccentric knee extension even in the same training status [22]. These studies suggest that both aerobic exercise-induced oxidative stress and anaerobic exercise-induced oxidative stress are attributed not only to the training status of the individual but also to interindividual variability. Therefore, focusing on the exercise type, intensity, and training status and considering wide variability of individuals such as reductive stress may provide better insight into exercise-induced oxidative stress following benefits and/or consequences. The aim of the present study was to establish an understanding of the effect of different types of exercise on oxidative stress.

## 2. Methodology

In accordance with the guidelines for the Preferred Reporting Items for Systematic Reviews and Meta-Analyses (PRISMA) statement, a search for relevant articles was carried out on PubMed/Medline, ISI Web of Science, and Google Scholar terms using a broad range of synonyms including ROS, oxidative damage, oxidants, physical activity, aerobic exercise, and strength exercise until 2019. To avoid the risk of missing relevant articles, additional papers were searched on the grey literature (i.e., generic web search) and through the bibliography of previous reviews. One author (AT) ran the search and screened the initial titles after duplicates were removed. Two authors (AT and GY) independently examined potentially relevant articles in depth. We included only papers published in peer-reviewed journals which reported findings from experimental controlled studies, i.e., human studies only. We excluded articles not available in English, unpublished papers, and conference posters or those reporting findings of nonexperimental studies (e.g., pre- and postintervention studies and case series). The first author's name, year of publication, sample of intervention and control group, design and duration of the study, topic and type of intervention, outcome, assessment, and results were recorded using an electronic spreadsheet.

## 3. Results

### 3.1. Search Results

**Figure 1:**
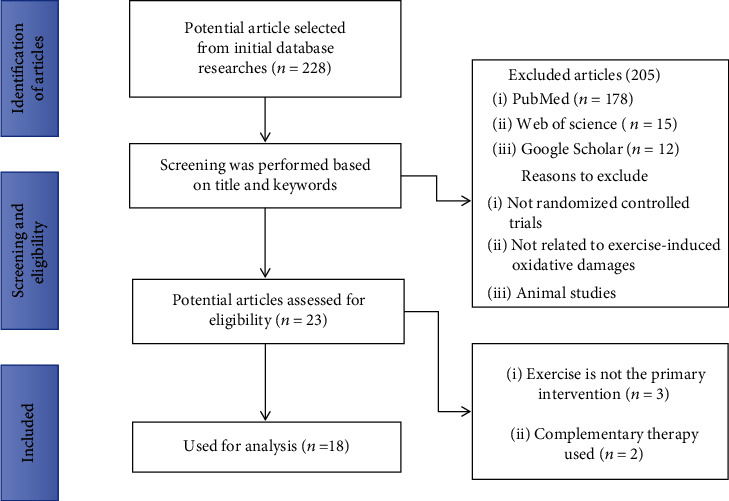
The search flowchart for the screening process.

Two hundred twenty-eight articles were identified from initial database searches and are presented in Figure 1. After screening was performed using titles and relative keywords, a total of 205 articles were excluded. The remaining 23 potential articles' abstracts were carefully evaluated, and five articles were excluded. The full text of the remaining 18 articles was retrieved and reviewed, which was then included for systemic analysis (Figure 1).

### 3.2. Participant Characteristics

The number of studies included for review was 18. The study population, the number of participants, mean age, intervention, and main outcomes are outlined in Table 1.

### 3.3. Study Selection

This study selected 18 articles for assessing the effect of different exercise protocols on reducing oxidative damage. A total of 17 articles were identified by searching databases, and one was identified by the article's reference for this study. All the records used in this study were on human subjects. Seven studies analyzed the effect of resistance exercise on oxidative damage, and eight studies analyzed the effect of aerobic exercise on oxidative damage. One study analyzed the combined effects of both aerobic and resistance training on oxidative damage. Two studies analyzed the general physical activity outcomes with the aim of reducing oxidative damage.

### 3.4. Risk of Bias of Included Studies

**Figure 2:**
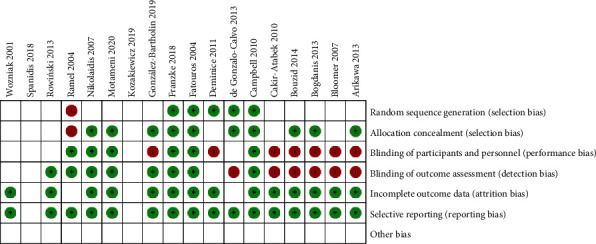
Risk of bias evaluation of the included studies.

From the 18 included studies, at least nine studies had a risk of bias. Eight studies had high risk of blinding of participants and outcome assessments (Table 2). One study had a high risk of randomization, and one study had allocation concealment. All the studies included in this study had a low risk of an incomplete outcome. A total of 12 studies had unclear risk in the randomization of participants, and all the 18 included studies had unclear risks associated with other bias (Figure 2).

### 3.5. Effect of Exercise on Oxidative Stress Markers

We selected a total of eighteen studies for the analysis of exercise effects on reducing oxidative damage. Selected studies used different oxidative damage markers such as malondialdehyde (MDA), protein carbonyl (PC), and isoprostanes and the level of enzymatic antioxidants like superoxide dismutase (SOD), catalase (CAT), total antioxidant capacity (TAC), and glutathione (GSH). Different training sessions were performed by the participants, and most of the studies that we selected belong to the regions of Europe-based countries. Four studies were from Greece, three from Poland, two from Austria, and one from France and Spain. There were three studies from the USA, one from Chile, and one from Brazil. There were also two studies from Middle East countries, Iran, and Turkey. One study used incremental exercise to perform moderate concentric and high-intensity eccentric exercise to analyze the oxidative damage [23]. The study found that moderate-intensity exercise did not increase oxidative stress. However, high-intensity exercise increased oxidative stress and muscle damage. Well-trained individuals were able to cope with the oxidative stress easily during eccentric exercise [41]. A further study found that no resistance training increased oxidative stress with the participants [24]. In contrast, other studies found that different combinations of resistance training combat the oxidative damages than usual resistance training [25, 34], whereas another study observed that circuit training decreased the oxidative damage after exercise [36]. Also, a study found that endurance training decreased oxidative stress and increased antioxidant status [27]. One session of high-intensity training (HIT) increased the oxidative stress along with antioxidants whereas a short session decreased the oxidative stress-induced damage along with improving antioxidants [29]. A further study also found that repeated bouts of exercises increased the oxidative stress-induced damage more than the first bout [31]. Different training modalities like running, ski running, and football playing increase the ROS generation along with antioxidants [32] whereas other studies found that increased maximal O_2_ uptake decreased the F2-isoprostane, a marker of oxidative damage [33, 35]. Other studies found that even low levels of physical activity such as fishing, long walking, team games, and picking mushrooms can reduce the oxidative stress-induced damage, but oxidative stress-induced damage was intensified with aging [38]. Another study found that both aerobic and resistance exercises increased the oxidative stress-induced damage [39], while one study found that resistance training minimally affected DNA oxidation and lipid profiles [28]. In one study, we analyzed only resistance training effects on oxidative stress which had other exercise components including cognitive ability upregulation [34]. In another study, we included only active young individuals in the analysis [30]. Most of the studies did not use any control groups; instead, they used pre- and postexercise values for analyzing the effect of exercise on oxidative damage [23, 24, 27–29, 31, 32, 36–38].

## 4. Discussion

Exercise-induced oxidative stress is an important factor in deciding both benefits and consequences including regulating signaling pathways or acting as a signaling molecule, biogenesis, and causing damages to the cells [11]. However, the way how exercise performance could affect the above-mentioned condition is ambiguous. Therefore, additional attention is necessary while prescribing exercise to the individual. This study systematically analyzed the different exercise training effects on oxidative stress condition, and we observed that any type of exercise can increase or decrease oxidative damage according to the exercise type, intenstiy and training status, and specific oxidative damage markers that are used to measure the oxidative damage. This study analyzed a total of 18 articles that focused on effect of exercise on reducing oxidative damage. Eight articles were selected for the effect of resistance training on oxidative stress biomarkers such as MDA and 8-hydroxydeoxyguanosine along with different antioxidant levels. Studies have shown that resistance training promotes muscle performance by reducing oxidative stress-induced consequences [6]. However, this study found that resistance training had no effect on reducing oxidative stress, whereas different combinations of resistance exercise had good effect on combating oxidative stress-related damages, and this could be due to specific oxidative damage markers and training status of the individual. Lima et al. [41] have shown that a single session of combined exercise reduced oxidative stress. Gomes et al. [42] have also shown that aerobic exercise is more superior in combating oxidative stress than resistance-type exercise. Further, some studies observed that well-trained exercise condition can even overcome high-intensity-induced oxidative damage in both aerobic and resistance exercise [43, 44].

### 4.1. Exercise Intensity and Oxidative Stress

Although increased intensity of exercise shifts the redox balance in favor of oxidative stress, this situation is necessary for initial adaption, supporting the concept of exercise-induced hormesis. Higher intensity exercise increases the total antioxidant capacity when compared to low and moderate intensities of treadmill exercise with no changes in lipid peroxidation [45]. Previously, it was believed that exercise with increased intensity must be overwhelmed by antioxidant defenses which induce a condition of oxidative stress [46]. However, recent reports suggested that even low or moderate intensity can induce oxidative stress, suggesting that exercise volume (duration × intensities) and failed antioxidant defense system are the primary mediators of exercise-induced oxidative stress. We found that different intensities of resistance training can effectively reduce the oxidative damage in the participants when compared to the same intensities. This could be due to exercise-induced redox-linked health adaptations through upregulating the antioxidant defense system [47]. Regarding aerobic exercise, moderate intensity improved the exercise performance by reducing oxidative damage [23]. However, short-term aerobic exercise with higher intensities can greatly induce oxidative damage, but this effect is not extended up to the DNA level [29]. The exercise type is an important factor for inducing oxidative damage because high intensity of cycling reduces the oxidative damage and increases the enzymatic antioxidants [29], but the same intensity with sprint exercise increases oxidative damage [28]. Similarly, resistance trainings such as circuit resistance training reduced the oxidative damage and improved the antioxidant level when compared to traditional resistance training, suggesting that the exercise type along with a total volume of exercise is important for exercise-induced oxidative stress. Other studies have shown that exercise volume is not enough to exhaust antioxidants and increase oxidative stress [48]. The disparity between these studies is due to exercise protocol duration, exercise mode, and the oxidative damage markers analyzed [49–51]. In the current study, different intensities particularly higher intensities with different modes of exercise can greatly reduce oxidative stress by stimulating the plasma antioxidants and subsequent ROS scavenging activities.

### 4.2. Effect of Different Exercises in Aging

Aging is associated with increased formation of ROS subsequently causing oxidative modification of protein, lipid, and DNA. The reason may be due to defects in the mitochondrial electron transport chain (ETC) and changes in the enzymatic activities of ETC. Consequently, this increases the leakage of electrons and formation of superoxide anion [52]. Physical exercise has several health benefits, but higher intensity induces muscle oxygen flux which ultimately increases oxidative injury. Further, biochemical changes due to aging can facilitate the increase in the formation of ROS and decrease in the muscle repair and regeneration capacity in the aging community. Therefore, it is important to consider several aspects before advising exercise for aged people. Chronic exercise is an effective strategy for reducing age-induced oxidative stress. Even any kind of physical activity is linked with a reduced level of oxidative stress in the elderly such as gaming, fishing, and gardening. This study found that normal physical activity such as fishing or gardening and both combined training of aerobic and resistance training can increase the antioxidants and reduce the oxidative damage in the elderly. However, exercise-reduced oxidative damage in aging may depend on the type of exercise and intensities [30, 38, 39]. For example, single bout of exercise can abruptively exceed a certain intensity or duration resulting in increased production of ROS whereas regular exercise improves the antioxidant defense system with tolerable oxidative damage which in turn induces significant adaptions. However, this can be related to exercise intensity. Bouzid et al. [30] have shown that moderate to higher intensity regular exercise can help to maintain better antioxidants in the elderly. This may be due to different oxidative stress training adaptation that is necessary for older tissues to prevent age-related diseases and to retard the aging process [53]. This preventative effect of aging is achieved through the formation of ROS in part or full to alter the signaling pathways and/or cause molecular damage that can produce adaptive responses to withstand further stress [54]. This study also corroborates with the above statements that different exercise or physical activity can prevent oxidative damage and upregulate antioxidants.

### 4.3. Methodological Limitation

In addition to fixing the exercise type and intensity to prevent oxidative damage, measuring ROS in the biological system is more complicated since they are very short-lived and highly reactive. Assessing exercise-induced oxidative damage used different oxidative damage markers, and these oxidative damage markers can only be reflected on specific local oxidative stress, but exercise-induced physiological response can occur in the entire system. This questioned the suitability of those markers in assessing the oxidative damage in the exercise condition. Next, sample stability during exercise is an important factor to consider because there is a possibility of the sample to become more viable to oxidative damage. In this aspect, the urinary sample is more convenient even for collection and provide better redox balance for a longer period when compared to blood samples in the exercise condition. However, only some oxidative damage markers are standardized in animals and humans, and this has to be done in the exercised samples with different training status.

### 4.4. Future Directions

Although several studies have reported that any type of exercise can greatly increase oxidative stress in a type- and intensity-dependent manner, other aspects such as specific oxidative damage markers, reductive stress, and training status of the individual should also need additional research. For example, some promising markers are underway such as acrolein-lysine which will diversify the current parameters to measure the oxidative damage. Further, the status of antioxidant defense during or after exercise is varied which could conflict the measurement of oxidative stress. Therefore, some integrative approaches such as OXY-SCORE or oxidative-INDEX computed by subtracting antioxidative capacity from ROS levels/ROS-induced damage or oxidative stress index (OSI) are required for the measurement of oxidative stress. Taken together, the measurement of oxidative stress during different types of exercise needs reliable research work that should predict earlier oxidative damage in the exercise condition.

## 5. Conclusion

This systematic review observed that any type of exercise or physical activity can greatly increase or decrease oxidative damage in an exercise type and intensity manner and specific oxidative damage markers that are used to measure the oxidative damage. This could cause various misjudgment in predicting oxidative damage in the exercised condition. Further, the data analyzed in this study did not provide conclusive evidence related to the methodology to reduce and adapt to oxidative stress. This was partly due to the fact that studies reviewed did not complete follow-up evaluations. Therefore, different types of exercise using different intensities and durations need to be completed with follow-up programs, with large numbers to make full conclusions about reducing oxidative stress damage.

## Figures and Tables

**Table 1:** The study characteristics of included studies.

Reference	Study population	Mean age (years; E/C)	No. of participants (E/C)	Intervention	Assessment	Main results
González-Bartholin et al. [23]	Chile	60.4 ± 6.8	10	Ten healthy individuals participated in this study, and they performed a concentric cycling incremental test and moderate- and high-intensity concentric and eccentric cycling using a recumbent ergometer.	PCO MDA	Oxidative markers like PCO and MDA were analyzed, and moderate intensity of eccentric and concentric cycling did not increase oxidative stress and further muscle damage, but high-intensity exercise increases the OS and muscle damage.
Motameni et al. [24]	Iran	26.30 ± 4.95	10	All the participants performed the following exercise: hypertrophy type resistance exercise, strength, and power type resistance exercise.	H_2_O_2_ MDA	The study results observed that no resistance training increased OS.
Çakır-Atabek et al. [25]	Turkey	25.50 ± 4.72/29.00 ± 5.87	8/8	Intensity of resistance exercise consists of leg extension which is standardized for total volume: 1 × 17 reps at 50% of 1RM, 1 × 14 reps at 60% of 1RM, 1 × 12 reps at 70% of 1RM, 2 × 5 reps at 80% of 1RM, and 3 × 3 reps at 90% of 1RM with 5 minutes of rest between intensities and 90–120 seconds of rest between sets.	LHP PCO SOD	LHP, PCO, and SOD were increased during resistance exercise, and the study suggests that different combinations of intensities are more beneficial to combat oxidative stress.
Kozakiewicz et al. [26]	Poland	46/65	46/112	For the intervention group, the protocol consisted of moderate leisure-time physical activity (LTPA) 124 ± 09 METs × hour/week (or 744 ± 54 METs × min/week).	Isoprostane, PCO, MDA	The concentration of isoprostanes, PCO, and MDA decreased in the active subjects, but it depends on the aging condition.
Fatouros et al. [27]	Greece	72.8 ± 4.8	11/11	The endurance training group for 16 weeks consists of 3–5 min warmup, which consisted of light walking at approximately 40% of their maximal heart rate (attained during the exercise test), and 3–5 min warmup, which consisted of light walking at approximately 40% of their maximal heart rate (attained during the exercise test). Briefly, subjects walked/jogged at 50–80% of HRmax (0% grade) for 12–42 min each time (duration increased 2 min every week).	MDA, GPx, and TAC	Four months of endurance training decreased the oxidative stress and increased antioxidant status.
Bloomer et al. [28]	USA	24 ± 4	13	The exercise training session consists of sprint and squat tests (the sprint consisted of a standard 30 s Wingate test using a Monark cycle ergometer modified to conduct Wingate testing at a load equal to 7% of subjects' body weight) (the squat test consisted of performing 15 repetitions using a load equal to 70% of subjects' 1‐RM + bodyweight (system mass), using a Smith machine).	PCO MDA 8-oxodG	PCO was elevated following both squat and sprint exercise, whereas DNA oxidation and lipid were minimally affected.
Bogdanis et al. [29]	Greece	24.3 ± 1.4	8	Participants performed HIIT sessions, and the session consists of four to six 30 s bouts of high-intensity cycling.	PCO TBARS GPx	One session of HIT increased the oxidative stress along with increasing antioxidants, but a short session decreased the oxidative stress along with improving antioxidants.
Bouzid et al. [30]	France	21.4 ± 1.9/20.3 ± 2.8	16/15	Participants performed the incremental exercise test until exhaustion on an electrically braked cycle ergometer.	MDA SOD GPx GR	Regular physical exercise decreases lipid peroxidation, but it depends on aging condition.
Nikolaidis et al. [31]	Greece	23 ± 2	12	Isokinetic exercise session consists of 75 lengthening knee flexions, which were repeated after 3 weeks.	TAC TBARS PCO GSH CAT	Repeated bout of lengthening contractions induced much less muscle damage and blood oxidative stress than the first bout.
Wozniak et al. [32]	Poland	23.8 ± 2.9	20	Running, ski running, power-house, and football.	TBARS SOD CAT	Increased the ROS generation after the exercise along with increasing SOD and CAT.
Campbell et al. [33]	USA	60.7 ± 6.7/60.6 ± 6.8	87/86	All participants were allowed to perform maximal graded treadmill.	F2-isoprostane	Exercisers increased maximal O_2_ uptake, and F2-isoprostane decreased in exercisers, suggesting that aerobic exercise, when accompanied by relatively marked gains in aerobic fitness, decreases oxidative stress.
Franzke et al. [34]	Austria	(65–83)	41/40	Resistance training consists of elastic bands, chairs, and own body weight.	GSH 8-oxo-dG MDA CAT SOD	Six months of elastic band resistance training improved physical function, as well as overall redox status. Better fitness level was linked to reduced oxidative DNA damage.
Arikawa et al. [35]	USA	18-30	166/153	16-week aerobic exercise intervention consists of 30 minutes of weight-bearing aerobic exercise, five times per week, at a specified intensity based on age-predicted heart rate maximum (HRmax). Exercise intensity increased every four weeks to reach 80–85% of HRmax.	F2-isoprostanes	Benefits of aerobic exercise in reducing systemic oxidative stress may be limited to those who present higher baseline levels of plasma F2-isoprostanes.
Deminice et al. [36]	Brazil	25.9 ± 2.8	11	Hypertrophy-resistance traditional interval training (3310 repetitions at 75% of the 1 repetition maximum (1RM), with 90-second passive rest) and hypertrophy-resistance circuit training (3310 repetitions at 75% of the 1RM, in alternating performance of 2 exercises with different muscle groups) in 2 different weeks.	TBARS GSH MDA	Circuit resistance hypertrophy training lowered the oxidative stress and antioxidants when compared to resistance training.
Ramel et al. [37]	Austria	28.2 ± 3.9/31.3 ± 10.2	17	Resistance exercise circuit (bench press, leg press, latissimus dorsi pull, leg extension, shoulder press, triceps exercise, crunch, vertical row, biceps curl and pull up), and their one-repetition maximum (1RM) for each exercise.	MDA Ascorbic acid	Plasma MDA and CD concentrations increase after exercise.
Rowiński et al. [38]	Poland	65–69	481	Long walking, gardening, fishing, picking mushrooms, swimming, skiing, team games.	Isoprostanes SOD GPx CAT	Physical activity resulted in a decrease in plasma iso-PGF2*α* and protein carbonyl concentration, but OS is intensified with aging.
de Gonzalo-Calvo et al. [39]	Spain	79 ± 5/74 ± 5	13/13	Training was self-directed and combined endurance and resistance activities.	MDA 4-HNE SOD TAC	Results indicate that chronic exercise from middle age to old age increases oxidative damage; however, chronic exercise appears to be an effective strategy to attenuate the age-related decline in the elderly.
Spanidis et al. [40]	Greece	22.5 ± 0.58	22/18	An eccentric exercise session was performed on an isokinetic dynamometer, and exercise protocols were undertaken from the seated position (120° hip angle) with the lateral femoral condyle aligned with the axis of rotation of the dynamometer.	PCO MDA TAC CAT	Trained individuals are less susceptible to oxidative damage.

Note: E: experimental group; C: control group; PCO: protein carbonyl; TBARS: thiobarbituric acid reactive substances; MDA: malondialdehyde; TAC: total antioxidant capacity; GR: glutathione reductase; SOD: superoxide dismutase; GPx: glutathione peroxidase; CAT: catalase; GSH: glutathione; 4-HNE: 4-hydroxynonenal; LHP: lipid hydroperoxide; ROS: reactive oxygen species; H_2_O_2_: hydrogen peroxide; 8-oxodG: 8-hydroxydeoxyguanosine; 8-oxo-dG: 8-oxo-2′-deoxyguanosine.

**Table 2:** Risk of bias evaluation of included studies.

Reference	Random sequence generation	Allocation concealment	Blinding of participants and personnel	Blinding of outcome assessment	Incomplete outcome data	Selective reporting	Other bias
Calabrese and Baldwin [12]	Unclear	Low	High	Low	Low	Low	Unclear
Dillard et al. [13]	Unclear	Low	Low	Low	Low	Low	Unclear
Lovlin et al. [14]	Unclear	Unclear	High	High	Low	Low	Unclear
Powers et al. [5]	Unclear	Unclear	Unclear	Unclear	Unclear	Unclear	Unclear
Quindry et al. [15]	Low	Low	Low	Low	Low	Low	Unclear
Alessio et al. [16]	Unclear	Unclear	High	High	Low	Low	Unclear
Bloomer et al. [17]	Unclear	Low	High	High	Low	Low	Unclear
Marzatico et al. [18]	Unclear	Low	High	High	Low	Low	Unclear
McBride et al. [19]	Unclear	Low	Low	Low	Low	Low	Unclear
Hoffman et al. [20]	Unclear	Unclear	Unclear	Unclear	Low	Low	Unclear
Bloomer et al. [21]	Low	Low	Low	Low	Low	Low	Unclear
Margaritelis et al. [22]	Low	Low	Low	Low	Low	Low	Unclear
González-Bartholin et al. [23]	Unclear	Low	High	High	Low	Low	Unclear
Motameni et al. [24]	Low	Unclear	High	Unclear	Low	Low	Unclear
Çakır-Atabek et al. [25]	High	High	Low	Low	Unclear	Low	Unclear
Kozakiewicz et al. [26]	Unclear	Unclear	Unclear	Low	Low	Low	Unclear
Fatouros et al. [27]	Low	Low	Unclear	High	Unclear	Low	Unclear
Bloomer et al. [28]	Unclear	Unclear	Unclear	Unclear	Unclear	Unclear	Unclear
